# Targeted Delivery of Secretory Promelittin via Novel Poly(lactone‐*co*‐β‐amino ester) Nanoparticles for Treatment of Breast Cancer Brain Metastases

**DOI:** 10.1002/advs.201901866

**Published:** 2020-01-19

**Authors:** Yu Zhou, Shenqi Zhang, Zeming Chen, Youmei Bao, Ann T. Chen, Wendy C. Sheu, Fuyao Liu, Zhaozhong Jiang, Jiangbing Zhou

**Affiliations:** ^1^ Department of Neurosurgery Yale University New Haven CT 06511 USA; ^2^ Department of Neurosurgery The Second Xiangya Hospital of Central South University Changsha Hunan 410011 China; ^3^ Department of Neurosurgery Renmin Hospital of Wuhan University Hubei 430060 China; ^4^ Department of Biomedical Engineering Yale University New Haven CT 06511 USA

**Keywords:** brain metastasis, breast cancer, gene therapy, melittin, poly(lactone‐*co*‐β‐amino ester)

## Abstract

Breast cancer brain metastases (BCBM) is a devastating disease with dismal prognosis. Although chemotherapy is widely used for clinical management of most tumors, it is often ineffective for BCBM. Therefore, alternative approaches for improved treatment of BCBM are in great demand. Here, an innovative gene therapy regimen is reported that is designed for effective treatment of BCBM. First, poly(lactone‐*co*‐β‐amino ester) nanoparticles that are capable of efficient gene delivery are synthesized and are engineered for targeted delivery to BCBM through surface conjugation of AMD3100, which interacts with CXCR4 enriched in the tumor microenvironment. Next, an artificial gene, *proMel*, is designed for the expression of secretory promelittin protein, which has limited toxicity on its own but releases cytolytic melittin after activation by MMP‐2 accumulated in tumors. It is demonstrated that delivery of the *proMel* via the AMD3100‐conjugated nanoparticles effectively inhibits tumor progression in a BCBM mouse model. This study suggests a new direction to treat BCBM through targeted delivery of promelittin‐mediated gene therapy.

## Graphical abstract

Herein, a new generation of poly(lactone‐*co*‐β‐amino ester) is synthesized for gene delivery and an artificial gene, proMel, is designed for effective cancer gene therapy. The targeted delivery of proMel effective treatment of breast cancer brain metastases is demonstrated.

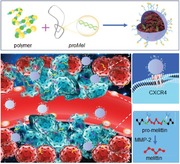

## 1 Introduction

Breast cancer is the second leading cause of cancer‐related mortality in women, with over 41 000 deaths annually in the United States alone.^1^ Of these patients, 15–30% eventually develop breast cancer brain metastases (BCBM).^2^ Current treatments for BCBM are palliative. Standard therapy involves surgical resection, if possible, followed by radiation therapy. Because chemotherapy is usually ineffective for BCBM, it is excluded in the standard of care.^3^ This lack of effective pharmacological therapies is largely due to the resistance of BCBM to chemotherapeutic agents,^4^ and the existence of the blood‐brain barrier (BBB), which prohibits adequate drug delivery to the brain.^5^ As a result, the prognosis for BCBM patients has been dismal with a median survival of 2.3–7.1 months.^6^ Thus, development of alternative strategies for treatment of BCBM is in great demand.

Gene therapy represents a promising alternative strategy for BCBM treatment. Different from traditional chemotherapy which requires multiple drug administrations for long‐term efficacy, gene therapy can produce a persistent therapeutic benefit after one single successful transfection. However, development of systemic gene therapy for BCBM has been largely limited by the inability to safely deliver genetic materials to metastatic tumors in high efficiency. In current clinics, gene therapy is often administered using engineered viral vectors, which have been shown to be unsafe for systemic applications.^7^ Nonviral vectors have emerged as a safer alternative; however, they have been limited by their low efficiency and high toxicity.[qv: 5b,8] Recently, several nonviral nanoparticles (NPs), including a family of poly(amine‐*co*‐ester) terpolymer NPs synthesized by our group, have demonstrated a great capacity for delivery of gene therapy with high efficiency and safety.^9^ Thus, delivery of cancer gene therapy via nonviral vectors is potentially feasible. Development of systemic gene therapy for BCBM is further limited by the lack of effective genetic materials. Typically, gene delivery vectors target tumor cell survival; however, it is unlikely that systemic administration of these vectors successfully transfects all tumor cells. As a result, nontransfected tumor cells can continue to propagate and eventually kill the patient. This limitation can be potentially overcome through delivery of suicide genes, such as cytosine deaminase and herpes simplex virus thymidine kinase, which convert prodrugs to their active forms and kill nontransfected tumor cells through a bystander effect.^10^ Nevertheless, the efficacy of cancer suicide gene therapy has shown to be limited by two main reasons.^10^, ^11^ First, since the killing effect of cancer suicide gene therapy depends on the active forms of pharmacological agents, tumor cells may develop drug resistance as a result of genetic and epigenetic instability. Second, the products of suicide genes are cytosolic enzymes, which cannot be dispersed over a long distance to kill distal tumor cells. As a result, the bystander effect is limited.

Here, we report a novel nonviral gene therapy approach that can overcome the aforementioned hurdles for effective BCBM treatment. First, in order to improve gene delivery to BCBM, we synthesized a family of poly (PDL‐*co*‐MDEA‐*co*‐TDDP) (PPMTP) polymers through polymerization of ω‐pentadecalactone (PDL), *N*‐methyldiethanolamine (MDEA) with diethyl 3,3′‐(4,4′‐trimethylenedipiperidine‐1,1′‐diyl)dipropionate (TDDP) with and without poly(ethylene glycol) (PEG). By screening those polymers, we identified a PEGylated polymer, PEG30%P, which consists of 30% PDL by mole, which can form solid NPs and deliver genes with high efficiency and favorable pharmacokinetic (PK). We further engineered PEG30%P NPs through surface conjugation of AMD3100, a small molecule antagonist of CXCR4 that is known to be highly expressed in BCBM.^12^ and demonstrated that the resulting AMD3100‐conjugated PEG30%P NPs, or AP30NPs, enables targeted gene delivery to BCBM. Next, to improve the efficacy of gene therapy, we designed an artificial gene, *proMel*, for production of secretory promelittin. Melittin is a water‐soluble cytolytic peptide derived from bee venom of *Apis mellifera* that has significant cytotoxicity.^13^ Application of this peptide for cancer treatment has been limited by its nonspecific toxicity to normal cells. Unlike melittin, promelittin has minimal cytotoxicity in normal physiological conditions but can be activated by matrix metalloproteinase 2 (MMP‐2) that is enriched in the tumor microenvironment.^14^ This activation triggers release of melittin, which, in turn, kills surrounding tumor cells. Lastly, we demonstrated that targeted delivery of *proMel* via AP30NPs effectively inhibits BCBM development.

## 2 Results

**Figure 1:**
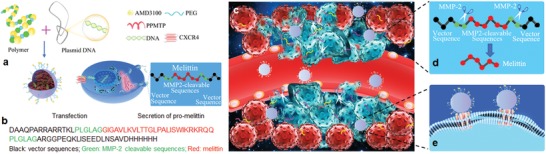
Targeted delivery of *proMel* gene for BCBM treatment. a) Schematic diagram of NP synthesis and *proMel* delivery. b) Sequence of promelittin protein. c) Schematic diagrams of targeted delivery of *proMel* via AP30NPs. e) After intravenous administration, the NPs accumulate preferentially in tumors through the interaction between AMD3100 and CXCR4 and transfected tumor cells. d) The transfected cells produce and secrete promelittin, which is activated by MMP‐2 and releases melittin to kill tumor cells.

Effective treatment of BCBM requires delivery of genetic materials to metastatic tumors and killing of both transfected and nontransfected tumor cells. To achieve this goal, we delivered proMel to BCBM via AP30NPs (**Figure** 1a). *proMel* is an artificial gene designed for expression of secretory promelittin protein, which consists of one copy of melittin flanked by PLGLAG, an MMP‐2 cleavable peptide, and sequences from pSecTag2 by Invitrogen, Thermo Fisher Scientific (Figure 1b). The vector contains a murine Ig κ‐chain leader sequence right ahead of the gene of interest, which allows secretion of the fusion protein after expression. After successful delivery, transfected cells produce and secrete promelittin, which diffuses into surrounding tissues (Figure 1a,c). Promelittin is not toxic to normal tissue but can be activated by MMP‐2 that is enriched in tumor microenvironment, leading to release of free melittin, which lyses and kills adjacent tumor cells (Figure 1d). *proMel* was delivered via AP30NPs, which were designed for efficient gene delivery specifically to metastatic tumors. After intravenous administration, AP30NPs selectively accumulated in BCBM through the interaction between AMD3100 and CXCR4 and transfected tumor cells (Figure 1e). *proMel*‐loaded AP30NPs were assessed for BCBM treatment using mouse xenografts established through intracardiac injection of MDA‐MB‐231‐Br‐HER2 (231BR) cells, a model that has been well‐characterized to recapitulate pathology of human BCBM.^15^

### 2.1 Synthesis and Characterization of PPMTP and PEG‐PPMTP

**Scheme 1:**
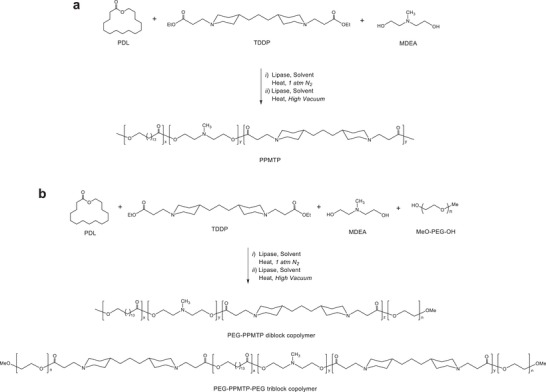
Enzyme‐mediated copolymerization for synthesis of a) PPMTP terpolymers and b) PEG‐PPMTP block copolymers.

We recently synthesized a family of terpolymers through polymerization of diethyl sebacate (DES), MDEA, and lactones through enzyme‐catalyzed polymerization chemistry. This synthetic approach is unique in that it allows for tuning three parameters, which are important for nonviral gene delivery, in a single molecule: positive charge, molecular weight, and hydrophobicity. As a result, polymers in this family, including terpolymers consisting of DES, MDEA, and PDL, are capable of delivering gene therapy in unprecedented efficiency.[qv: 9a] Among the three monomers, MDEA, which is weakly basic and protonated at acidic pH, contributes significantly to the gene delivery ability of the terpolymers by enhancing the encapsulation of genetic materials through electrostatic interaction and promoting endosomal escape through a proton sponge effect.[qv: 9a,16] We hypothesized that incorporation of additional amino groups with stronger basicity in the polymer chain can further enhance its delivery efficiency. To test this hypothesis, we replaced DES with TDDP, which consists of piperidine structures and has strong basicity, and synthesized a family of PPMTP via CALB lipase‐catalyzed copolymerization (**Scheme** 1a). Synthesis and characterization of TDDP are described in the Supporting Information (Scheme S1, Figure S1). Composition of PPMTP polymers was varied by adjusting the monomer ratio in the feed mixtures (**Table** 1). The copolymers were obtained in comparable yields (80–84%) with molecular weights (*M*_w_) ranging from 18 900 to 22 600 Da and polydispersity between 2.3 and 2.9 (Table 1).

**Table 1:** Characterization of PPMTP and PEG‐PPMTP copolymers

a					
Sample	PDL/TDDP/MDEA (feed molar ratio)	Isolated yield	PDL Content [mol%]^a)^	*M*_w_ [Da]	*M*_w_/*M*_n_
0%P	0:100:100	83%	0%	20700	2.4
10%P	10:90:90	80%	10%	20900	2.3
20%P	20:80:80	84%	21%	21600	2.5
30%P	30:70:70	83%	31%	22600	2.7
40%P	40:60:60	82%	41%	18900	2.9
b					
Sample	PDL/TDDP/MDEA/PEG (feed molar ratio)	Isolated yield	PDL Content [mol%]^a)^	*M*_w_ [Da]	*M*_w_/*M*_n_
PEG0%P	0:100:97:6	80%	0%	26200	1.7
PEG10%P	10:90:87.2:5.5	82%	11%	25300	1.8
PEG20%P	20:80:77.4:5.2	83%	21%	25900	1.8
PEG30%P	30:70:67.5:4.9	83%	30%	26700	1.8
PEG40%P	40:60:57.7:4.7	85%	41%	27300	1.7

^a)^ Mol% PDL units versus (PDL + TDDP) units in the copolymer chains.

We previously learned that poly(lactone‐*co*‐β‐amino ester) terpolymer NPs are generally unstable, making it difficult for in vivo applications.[qv: 9a,16] To overcome this limitation, we tuned the enzyme‐catalyzed chemistry and synthesized PEGylated PPMTP, or PEG‐PPMTP, by including MeO‐PEG5K‐OH as the chain terminator (Scheme 1b). By varying the monomer feed ratio, PEG‐PPMTP copolymers with different compositions were obtained (Table 1). During the copolymerization reactions, calculated amounts of MeO‐PEG5K‐OH were employed to form polymer products with approximately 40 wt% PEG upon complete feed conversion. The synthesis of PEG‐PPMTP with the controlled PEG content (40 wt%) was primarily based on our earlier results showing that at this PEG content, similar PEG‐poly(amine‐*co*‐ester) block copolymers readily undergo self‐assembly in aqueous medium to form micelle nanoparticles with high colloidal stability.^17^ The isolated yield for all copolymers ranged from 80% to 85%, and the polymer *M*_w_ varied from 25 300 to 27 300 Da with a polydispersity between 1.7 and 1.8 (Table 1). Since MeO‐PEG5K‐OH can only link to the ends of PPMTP chains, the PEG‐PPMTP copolymers likely contain two types of block chains, PEG‐PPMTP diblock chain and PEG‐PPMTP‐PEG triblock chain (Scheme 1b).

The molecular structures of PPMTP and PEG‐PPMTP copolymers were analyzed by both ^1^H and ^13^C NMR spectroscopy. The structural assignments for the proton and major carbon‐13 resonance absorptions of the copolymers without PDL (0%P, PEG0%P) and with PDL (40%P, PEG40%P) are delineated in Figures S2 and S3 (Supporting Information), respectively. Furthermore, the repeat unit distributions in the PPMTP chains or PPMTP segments of PEG‐PPMTP copolymers were quantitatively measured. As depicted in Figure S4a (Supporting Information), PPMTP and PEG‐PPMTP copolymers exhibit four carbonyl carbon‐13 resonances due to the presence of PDL*‐PDL, PDL*‐MDEA, TDDP*‐PDL, and TDDP*‐MDEA diads in the polymer chains. The measured abundances of the carbonyl carbon‐13 absorptions for 40%P, PEG20%P and PEG40%P are shown in Figure S4b (Supporting Information) and are compared to the corresponding values calculated for random PPMTP chains with same compositions. The results indicate that the distributions of PDL, MDEA and TDDP units in the PPMTP chains or PPMTP chain blocks are nearly statistically random. Proton and carbon‐13 NMR spectroscopy analyses revealed that the PPMTP blocks in PEG‐PPMTP copolymers possess identical chain structures as PPMTP copolymers. For example, compared to those of PPMTP, the ^1^H NMR spectra of PEG‐PPMTP are essentially identical except for an additional resonance at 3.6 ppm due to the presence of ethylene oxide (EO) units (Figures S2 and S3, Supporting Information). Consistently, excluding carbon‐13 absorption of EO units at 70.5 ppm, major ^13^C resonances between PEG‐PPMTP and PPMTP are substantially same.

### 2.2 Synthesis and Characterization of PPMTP and PEG‐PPMTP NPs

**Figure 2:**
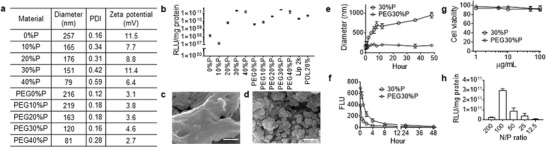
Characterization of PPMTP and PEG‐PPMTP NPs for gene delivery. a) Physical properties of the indicated NPs. b) Gene delivery efficiency of the indicated polymers on 231BR cells. Representative SEM images of c) 30%P NPs and d) PEG30%P NPs. e) Change of the diameter of the indicated NPs versus time in serum‐containing medium. f) Plasma concentrations of IR780 versus time in mice after intravenous administration of IR780‐loaded 30%P or PEG30%P NPs. g) Cytotoxicity of 30%P or PEG30%P NPs on 231BR cells. Toxicity was given as the percentage of viable cells remained after treatment for 3 d, compared to the control vehicle treated cells. h) The effect of 30%P NPs to DNA ratio on transfection efficiency on 231BR cells.

PPMTP and PEG‐PPMTP NPs were synthesized through a standard nanoprecipitation process as described in Methods. The resulting NPs were characterized for hydrodynamic diameter and zeta potential by a ZetaSizer. Results in **Figure** 2a show that PPMTP and PEG‐PPMTP NPs were in sizes ranging from 80 to 250 nm and bear positive surface charges. Inclusion of PEG reduced both the polydispersity and surface charge. We characterized both PPMTP and PEG‐PPMTP NPs for gene delivery by using plasmid DNA for expression of luciferase as a model gene and testing in 231BR cells. Lipofectamine 2000 (Lip 2k) and PDL20%, a terpolymer consisting of 20% PDL and 80% DES/MDEA by mole that demonstrated the greatest delivery efficiency in our previous study,[qv: 9a] were included as a control. 24 h after treatment, the cells were collected, and the expression of luciferase in cells were quantified. We found that 30%P and PEG30%P transfected 231BR cells in a comparable efficiency, which is significantly greater than other polymers and is 14 times greater than Lip 2k (Figure 2b). A similar trend was observed in human glioma U87MG cells (Figure S5a, Supporting Information). Based on these findings, we selected 30%P and PEG30%P NPs for further characterization.

First, we characterized the selected NPs for their stability. We found that both 30%P and PEG30%P NPs appeared to be spherical in shape and in nanosize when imaged by transmission electron microscopy (TEM) (Figure S5b,c, Supporting Information). However, only PEG30%P NPs, but not 30%P NPs, maintained their NP morphology after lyophilization, as revealed by scanning electron microscope (SEM) (Figure 2c,d). We further determined the stability of NPs in PBS containing 10% fetal bovine serum (FBS) by dynamic light scattering (DLS). Results in Figure 2e showed that PEG30%P NPs were stable in the solution without significant change of hydrodynamic diameter over 48 h; in contrast, 30%P NPs significantly aggregated over time. Those results suggested that, compared to 30%P NPs, PEG30%P NPs have greater stability. Next, we determined the PK of NPs in mice after intravenous administration. Results in Figure 2f showed that, compared to 30%P NPs, PEG30%P NPs had a significantly greater half‐life in the circulatory system. The enhancement of stability and PK in PEG30%P NPs could be attributed to the stealth effect resulting from PEGylation.^18^ Lastly, we evaluated the cytotoxicity of PPMTP and PEG‐PPMTP NPs in 231BR cells and found that both of them exhibited limited cytotoxicity across all the tested concentrations (Figure 2g). Due to their excellent stability, PK, and safety profile, PEG30%P NPs were selected for further studies.

In the transfection study, DNA was added to PEG30%P polymer at a N/P ratio of 100:1. The ratio was chosen because it was found to be optimal for the terpolymers developed in our previous study.[qv: 9a] To determine if this ratio was also optimal for PEG30%P, we tested a range of polymer to DNA ratios. We found that PEG30%P efficiently loaded DNA across all the tested conditions (Figure S5d, Supporting Information), and the ratio of 100:1 allowed for the greatest efficiency (Figure 2h). We showed that, at ratio of 100:1, PEG30%P NPs protected encapsulated DNA from enzymatic degradation (Figure S5e, Supporting Information) and released 61% of the loaded DNA over the first 36 h (Figure S5f, Supporting Information).

### 2.3 Engineering PEG30%P NPs for Targeted Delivery to BCBM

**Figure 3:**
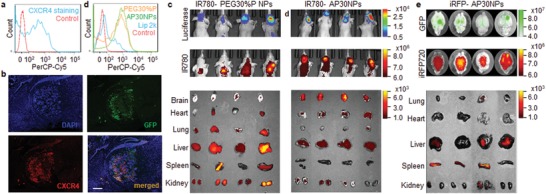
Characterization of A‐PEG30%P NPs for targeted gene delivery to BCBM. a) Flow cytometry analysis of the expression of CXCR4 in 231BR cells. b) Immunostaining analysis of the expression of CXCR4 in metastatic tumors in the brain. Scare bar: 500 um. c) IVIS imaging of tumors in the brain (top panel: luciferase signal), distribution of the indicated NPs in the brain (middle panel, IR780 signal), and in the indicated organs (bottom panel, IR780 signal). d) Flow cytometry analysis of the expression of iRFP720 in 231BR cells after treatment with the indicated NPs or Lip 2k. e) Ex vivo imaging of tumors in the brain (top panel: GFP signal), the expression of iRFP720 in the brain (middle panel, iRFP720 signal) and in the indicated organs (bottom panel, iRFP720 signal).

CXCR4 is a chemokine receptor known to highly express in MDA‐MB‐231 metastatic tumors.[qv: 12c,d] We confirmed that it is highly expressed in 231BR cells by flow cytometry (**Figure** 3a) and in metastatic tumors in the brain by immunostaining (Figure 3b). Compared to that in tumors, the expression of CXCR4 in normal brain tissues is significantly lower (Figure 3b, Figure S6, Supporting Information). The high expression of CXCR4 preferentially in tumors makes it a promising target for targeted drug delivery. We and others recently demonstrated that CXCR4‐targeted delivery can be achieved through surface conjugation of AMD3100.[qv: 12a,b] Following the same procedures used for synthesis of PEG30%P, except that maleimide‐PEG5K‐OH was used to replace MeO‐PEG5K‐OH in the monomer feed, we synthesized maleimide‐terminated PEG30%P, which possessed the same chain structures as PEG30%P. The presence of maleimide terminal groups is supported by a small distinct proton resonance of the polymer at 6.5 ppm. In contrast, all PEG‐PPMTP copolymers exhibited a small proton absorption at 3.4 ppm due to the methoxy (—OCH_3_) end groups. We utilized the same nanoprecipitation process as described above and synthesized maleimide‐terminated PEG30%P NPs, which were next subjected to conjugation of AMD3100 according to our recent report.[qv: 12a] The resulting AMD3100‐conjugated PEG30%P NPs, or AP30NPs, were spherical in morphology and had sizes comparable to unmodified NPs (Figure S7a, Supporting Information). Compared to 30%P NPs and PEG30%P NPs, AP30NPs demonstrated a slightly higher efficiency in gene delivery (Figure S7b, Supporting Information). This is likely due to the fact that surface conjugation of AMD3100 enhanced the uptake of AP30NPs, as a significant decrease of cellular uptake was observed when the expression of CXCR4 was down‐regulated in 231BR cells (Figure S7c,d, Supporting Information). We found that conjugation of AMD3100 did not alter the serum stability, cytotoxicity, PK of AP30NPs, which were comparable to those of PEG30%P NPs (Figure S7e–g, Supporting Information).

We evaluated AP30NPs for targeted gene delivery to BCBM. AP30NPs and control PEG30%P NPs were synthesized with encapsulation of IR780, a near‐infrared fluorescence dye that allows for noninvasive detection in live animals. The resulting NPs were intravenously administered to mice 3 weeks after intracardiac injection of green fluorescent protein (GFP)/luciferase—dually labeled 231BR cells.[qv: 9b] Imaging by in vivo imaging system (IVIS) showed that all the experimental mice had comparable tumor sizes (Figure 3c, upper panel). 24 h after NP administration, the mice were imaged and then euthanized. Major organs were isolated and further imaged ex vivo. We found that most PEG30%P NPs accumulated in peripheral organs, such as the liver; in contrast, significant amounts of AP30NPs accumulated in the brain (Figure 3c, middle and bottom panels). We quantified the uptake of NPs in tumor cells after intravenous administration. Experiment was carried out through the same procedures except NPs loaded with Rhodamine b (Rho B) were used. 24 h after NP administration, the mice were euthanized. The brains were isolated and digested. Single cells obtained from the digestion were analyzed by flow cytometry. 231BR tumor cells were identified based on the expression of GFP. We found that 52.4% of tumor cells were positive for NP uptake in mice treated with AP30NPs; in contrast, the efficiency was significantly lower in those treated with 30%P NPs and PEG30%P NPs (Figure S8a,b, Supporting Information).

We assessed AP30NPs for systemic delivery of genetic materials to BCBM. AP30NPs, together with control PEG30%P NPs, were synthesized with encapsulation of iRFP720, a plasmid DNA for expression of near‐infrared fluorescent protein,^19^ and evaluated in 231BR cells. Flow cytometry analysis showed that both NPs transfected 231BR cells in a comparable efficiency, which was significantly greater than Lip2k (Figure 3d). Next, we test iRFP720‐loaded AP30NPs in mice bearing 231BR BCBM. The mice were treated with the NPs daily for three consecutive days. 48 h later, the mice were euthanized. Major organs, including the brains, were harvested and subjected to IVIS imaging. We found that intravenous administration of iRFP720‐loaded AP30NPs efficiently transfected metastatic tumors in the brain, and, besides the brain, the livers and spleens were also transfected at various degrees (Figure 3e). We found that the area of iRFP720 signal is larger than that of GFP signal. This observation may not be a surprise. GFP emits fluorescence at 509 nm. With this emission wavelength, GFP fluorescence has a limited ability to penetrate brain tissue. Therefore, we can only detect those GFP signals emitted from the surface of the brain. In contrast, iRFP720 emits fluorescence at 720 nm. With this emission wavelength, iRFP720 fluorescence can penetrate brain tissue in a depth significant great than that of GFP. As a result, we can not only detect those iRFP720‐positive cells on the surface of the brain, but also those located within the brain. We quantified the transfection efficiency in tumors. Single cells were isolated from the brain and subjected to flow cytometry analysis. We found that 42.3% of tumor cells, which were identified based on GFP expression, were successfully transfected and expressed iRFP720 in mice treated with iRFP720‐loaded AP30NPs; in contrast, the efficiency was significantly lower in those treated with iRFP720‐loaded 30%P NPs and PEG30%P NPs (Figure S9a,b, Supporting Information). The finding was confirmed by confocal imaging analysis (Figure S9c, Supporting Information). We further analyzed the distribution of AP30NPs, which were identified based on the fluorescence of Rho B encapsulated in the NPs, and transfected cells, which were identified based on the expression of IR720, after intravenous administration, found that the NPs preferentially penetrated into tumors and transfected tumor cells but not normal cells in the brain (Figure S10, Supporting Information).

### 2.4 Production and Characterization of Promelittin

**Figure 4:**
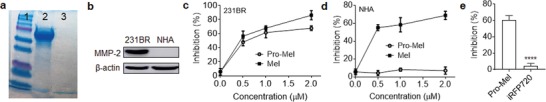
In vitro characterization of promelittin for cancer treatment. a) Analysis of cell supernatant before and after purification by SDS‐PAGE. 1) Marker; 2) Supernatant prior to purification; 3) promelittin eluted from the resin. b) Western blot analysis of MMP‐2 in 231BR and NHA cells. Growth inhibitory effect of promelittin on c) 231BR and d) NHA cells at the indicated concentrations. e) Growth inhibitory effects of AP30NPs loaded with *proMel* or iRFP720 on 231BR cells.

Melittin is a promising antitumor agent, whose application for cancer treatment has been limited by its significant nonspecific cytotoxicity.^13^ We recently found that the cytolytic effect of melittin can be abolished by flanking with short peptides.[qv: 12a] This finding inspired us to design *proMel*, an artificial gene for expression of an 8.4 kDa secretory promelittin protein as described in Figure 1b. To characterize its antitumor effect, we produced promelittin protein in CHO cells. Promelittin bears a His6 tag and thus allows for purification using Ni Superflow Resin (**Figure** 4a). The resulting promelittin was sensitive to MMP‐2, which efficiently cleaved promelittin at a dose‐dependent manner. For instance, after incubation with 100 × 10^−9^ m MMP‐2 at 37 °C for 30 min, 67.3% of promelittin was cleaved (Figure S11, Supporting Information). We characterized the cytotoxicity of promelittin in 231BR cells, which produce MMP‐2 (Figure 4b), and found that promelittin inhibited cell proliferation in efficiencies comparable to free melittin across all the tested concentrations (Figure 4c). In contrast, promelittin did not show toxicity to normal human astrocyte (NHA) cells, which produced less MMP‐2 (Figure 4b), in the same condition (Figure 4d). Next, we evaluated if delivery of *proMel* gene by AP30NPs kills 231BR cells. AP30NPs were synthesized with encapsulation of *proMel*. The resulting NPs, designated as *proMel* NPs, were added to 231BR cells. Control cells were treated with iRFP720‐loaded AP30NPs, or iRFP720 NPs. Results in Figure 4e show that treatment with *proMel* NPs inhibited cell proliferation by 60%; in comparison, treatment with iRFP720 NPs exhibited limited toxicity.

### 2.5 Targeted Delivery of *proMel* for Treatment of BCBM

**Figure 5:**
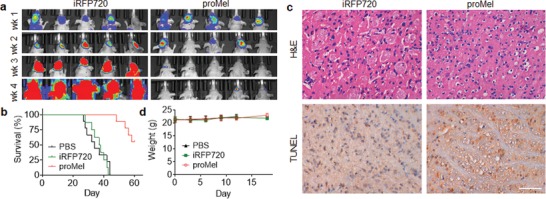
Characterization of targeted delivery of *proMel* for treatment of BCBM. a) Representative images of the development of tumors in the brain imaged by IVIS in mice received the indicated treatment. b) Kaplan–Meier survival curves of tumor‐bearing mice received the indicated treatments. c) H&E staining and TUNEL staining of tumors isolated from mice received the indicated treatments. Brown staining indicated TUNEL positive cells. Scale bar: 50 um. d) Change of body weight of mice received the indicated treatments with time.

We assessed *proMel* NPs for systemic treatment of BCBM. Mouse xenografts bearing BCBM were established through intracardiac injection of 231BR that were engineered to express luciferase. After 7 d, the mice were treated with *proMel* NPs through tail vein injection three times a week for two weeks. Control mice received treatments of iRFP720‐loaded AP30NPs or PBS. Intravenous administration of melittin killed mice and, thus, was not included as a control. The mice were monitored for development of tumors by IVIS imaging, body weight, and survival. Imaging of luciferase signal showed that treatment with *proMel* NPs significantly inhibited tumor progression (**Figure** 5a). Consistently, we found that treatment *proMel* NPs effectively enhanced the survival of tumor‐bearing mice (*p* < 0.0001) (Figure 5b). In contrast, treatment with *proMel*‐loaded 30%P NPs or PEG30%P NPs failed to achieve a comparable efficacy (Figure S12a, Supporting Information). The significant antitumor effect observed in the treatment groups is likely mediated by melittin released from promelittin, as promelittin without cleavage has limited cytotoxicity (Figure 4). Melittin is highly toxic and could not be given intravenously to generate antibodies. As a result, there are no commercial antimelittin antibodies available for detection and quantification of melittin within tumors.^20^ However, promelittin protein bears a His tag, which allows determining the expression of promelittin in tumors through isolation using Ni resin. Through this approach, we confirmed the expression of promelittin in tumors after intravenous delivery of *proMel* NPs (Figure S12b, Supporting Information). Histochemical analyses by hematoxylin and eosin (H&E) staining and terminal deoxynucleotidyl transferase dUTP nick end labeling (TUNEL) staining showed that treatment with *proMel* NPs reduced malignancy of the tumor phenotype and increased cellular apoptosis in tumors (Figure 5c). Further analysis of the H&E staining found that treatment with *proMel* NPs did not induce significant damage to normal brain tissue around tumors (Figure S12c, Supporting Information). This observation supports the hypothesis that promelittin could not be processed in normal tissues, which secret a limited amount of MMP‐2, and, thus, has limited cytolytic effects to the normal brain.

AP30NPs are potentially safe for intravenous applications. Throughout the animal studies, no loss of body weight was found in all the treatment groups, suggesting that the NPs have limited systemic toxicity (Figure 5d, Figure S13a, Supporting Information). The observed limited systemic toxicity may not be a surprise, as in vitro analyses found that the polymers have minimal cytotoxicity (Figure 2g, Figure S7g, Supporting Information), and hemolytic activity (Figure S13b,c, Supporting Information). In addition, PPMTP copolymers contain ester linkages, which are hydrolytically degradable. The biodegradability, which was confirmed by analyzing the change of molecular weight with time during incubation in PBS (Figure S13d, Supporting Information), further alleviates the concern of potential systemic side effects.

## 3 Discussion

Improved treatment of BCBM requires development of novel therapeutic regimens. In this study, we demonstrated that effective treatment of BCBM can be achieved through systemic delivery of *proMel*‐loaded AP30NPs. The significant antitumor effects of the *proMel* NP therapy can be attributed to the following two reasons.

First, the *proMel* NP therapy was delivered via novel AP30NPs, which allows for targeted delivery of genetic materials to metastatic tumors in high efficiency. AP30NPs were designed and synthesized based on our recent progress in developing novel polymers for gene delivery,[qv: 9a] and in engineered NPs for CXCR4‐targeting drug delivery.[qv: 12a] We found that the novel PEG30%P polymer is superior to the polymers developed in our previous study in that it not only can deliver genetic materials in greater efficiency but also have favorable PK due to the presence of PEG (Figure 2). We demonstrated that conjugation of AMD3100 enhanced the delivery of NPs preferentially to tumors with high efficiency (Figure 3).

Second, the antitumor effect of *proMel* NP therapy was mediated by secretory promelittin protein. Melittin, due to its significant cytolytic effect, is known as a potent antitumor agent. However, free melittin cannot be used for systemic cancer treatment due to its significant systemic toxicity. We demonstrated that the significant antitumor effect of melittin can be capitalized through delivery of *proMel*‐mediated gene therapy. We showed that, after successful delivery of *proMel* gene, transfected cells produce promelittin protein, which by itself has limited toxicity, but exhibits a cytolytic effect comparable to free melittin after activation by MMP‐2. As MMP‐2 is preferentially accumulated in tumors but not normal tissues, this feature avoids potential side effects of melittin to normal tissues and provides further tumor‐targeting specificity on top of the interaction between AMD3100 and CXCR4. The *proMel*‐mediated gene therapy has an additional benefit in that it can effectively kill surrounding tumor cells through a bystander effect. Promelittin protein after secretion is able to migrate to adjacent tumors cells, where it is activated and can kill nontransfected cells. The *proMel*‐mediated gene therapy is advantageous over the traditional suicide gene therapy in two aspects. First, it does not require administration of prodrugs. Second, promelittin is a secretory protein and can migrate over a long distance and kill distal tumor cells. In contrast, the enzymes produced in suicide gene therapy are cytosolic. As a result, the bystander effect of the suicide gene therapy may be limited to the regions adjacent to transfected cells. The *proMel*‐mediated gene therapy might be also superior to a recently developed suicide gene therapy using secretory TRAIL.^21^ TRAIL therapy, whose efficacy depends on the expression of death receptors DR4 and DR5 on target cells, has a major limitation in that some tumors lack expression of those receptors, and many tumors develop resistance toward TRAIL over time.^22^ In contrast, melittin kills cancer cells through cytolytic effects without selectivity. Consequently, the chance of development of resistance of the *proMel*‐mediated gene therapy is minimal.

Despite the significant efficacy observed in the study, the potential of *proMel*‐mediated gene therapy has not been fully capitalized. In addition to the lack of effective therapeutics, the existence of the BBB represents another major hurdle for BCBM treatment. However, AP30NPs described in this study were not designed to cross the BBB. Instead, they penetrated into BCBMs through BBB leakage and CXCR4‐mediated targeting. We found that 52.4% of tumor cells were positive for NP uptake in mice treated with AP30NPs (Figure S8, Supporting Information), and 42.3% of tumor cells were successfully transfected when iRFP720 DNA was delivered (Figure S9, Supporting Information). We demonstrated that, this degree of penetration, although may be insufficient for treatment of BCBMs through delivery of chemotherapy drugs, was adequate to produce significant therapeutic benefits through *proMel*‐mediated gene therapy (Figure 5). We speculate that further engineering the NPs for crossing the BBB, through approaches such as autocatalysis,[qv: 9b,23] would lead to a tumor‐inhibitory effect greater that what we observed in this study.

## 4 Conclusion

In summary, gene therapy for intracranial tumors, including BCBM, has been previously explored.[qv: 5b,24] However, clinical translation of cancer gene therapy has been limited by several hurdles, including the lack of safe gene delivery vehicles and effective therapeutic targets. We have developed a novel gene therapy regimen which can overcome all the major limitations associated with traditional gene therapy approaches. We demonstrated that this regimen effectively inhibits the progression of BCBM in the brain and prolongs the survival of tumor‐bearing mice. This novel approach may represent a new direction for effective treatment of BCBM.

## 5 Experimental Section

##### Cell Culture and Materials

NHA cells were obtained from the ATCC. 231BR cells were kindly provided by Prof. Patricia S. Steeg at the NCI. All cells were grown in Dulbecco's modified Eagle's medium (ThermoFisher) with 10% fetal bovine serum (ThermoFisher), 1% penicillin and streptomycin, and were maintained at 37 °C and 5% CO_2_. Maleimide poly(ethylene glycol) (MAL‐PEG‐OH, MW = 5000) was purchased from Jenkem Technology. AMD3100 octahydrochloride was purchased from Santa Cruz Biotechnology. Unless otherwise stated, all chemicals were purchased from Sigma‐Aldrich. Plasmid pGL4.13 for expression of luciferase was obtained from Promega. piRFP720‐N1 plasmid was a gift from Vladislav Verkhusha (Addgene plasmid # 45461).^19^ DNA for expression of promelittin, sequence of which was included in Figure S14 (Supporting Information), was synthesized by Gene Universal and cloned into pSecTag2 (ThermoFisher). For expression of GFP and luciferase, DNA for expression of enhanced green fluorescent protein (EGFP) and luciferase was amplified by polymerase chain reaction from pEGFPLuc (Clontech), and cloned into pCDH‐CMV‐MCS‐T2A‐Puro lentiviral vector (System Biosciences). Lentiviral particles production and cell transduction were carried out according to the previous reports.^25^

##### Polymer Characterization

^1^H and ^13^C NMR spectra were recorded on a Bruker AVANCE 500 spectrometer. The chemical shifts reported were referenced to internal tetramethylsilane (0.00 ppm) or to the solvent resonance at the appropriate frequency. The number and weight average molecular weights (*M*_n_ and *M*_w_, respectively) of polymers were measured by gel permeation chromatography (GPC) using a Waters HPLC system equipped with a model 1515 isocratic pump, a 717 plus autosampler, and a 2414 refractive index (RI) detector with Waters Styragel columns HT6E and HT2 in series. Empower II GPC software was used for running the GPC instrument and for calculations. Both the Styragel columns and the RI detector were heated and maintained at 40 °C temperature during sample analysis. Chloroform was used as the eluent at a flow rate of 1.0 mL min^−1^. Sample concentrations of 2 mg mL^−1^ and injection volumes of 100 µL were used. Polymer molecular weights were determined based on a conventional calibration curve generated by narrow polydispersity polystyrene standards from Aldrich Chemical Co.

##### Synthesis of PEG‐PPMTP

To prepare reaction mixtures, PDL, TDDP, MDEA, poly(ethylene glycol) methyl ether (5000 Da, MeO‐PEG5K‐OH), and Novozym 435 (immobilized *Candida antarctica* lipase B) catalyst were added to diphenyl ether solvent. The ratios of the reaction substrates are shown in Table 1. For all polymerization reactions, 10 wt% of the catalyst and 200 wt% of the solvent (vs total substrate) were used. The formed reaction mixtures were stirred at 90 °C under 1 atm pressure of nitrogen gas for 20 h during the first stage oligomerization, and subsequently under 1.8 mmHg vacuum for 70 h during the second stage polycondensation. At the end of the polymerization reactions, the product mixtures were added to *n*‐hexane to precipitate the resultant copolymers. The copolymers were then washed twice with *n*‐hexane and dissolved in chloroform. After filtrating the polymer solutions to remove the catalyst particles, the filtrates were evaporated at 30 °C under high vacuum (<1 mmHg) overnight to yield purified PEG‐PPMTP block copolymers. The PEG‐PPMTP copolymers are abbreviated as PEG*x*%P, in which *x*% indicates molar percentage content of PDL units versus (PDL + TDDP) units in the polymer chains. PEG‐PPMTP without PDL: ^1^H NMR (CDCl_3_; ppm) 1.18 (br., 10H), 1.27 (br., 2H), 1.65 (br., 4H), 1.94 (t/br., 4H), 2.34 (s, 3H), 2.51 (t, 4H), 2.66 (t, 4H), 2.70 (t, 4H), 2.86 (d/br., 4H), 3.64 (s, 28H), 4.17 (t, 4H), plus a singlet at 3.38 ppm; ^13^C NMR (CDCl_3_; ppm) 23.85, 32.31, 32.36, 35.60, 36.71, 42.87, 53.75, 53.86, 55.88, 62.13, 70.53, 172.57. PEG‐PPMTP with PDL: ^1^H NMR (CDCl_3_; ppm) 1.19 (br.), 1.25 (br.), 1.64 (br.), 1.94 (t/br.), 2.30 (m), 2.34 (s), 2.52 (t), 2.66 (t), 2.70 (t), 2.86 (d/br.), 3.64 (s), 4.06 (m), 4.17 (m), plus a singlet at 3.38 ppm; ^13^C NMR (CDCl_3_; ppm) 23.87, 24.91, 25.00, 25.89, 25.92, 28.60, 28.64, 29.13, 29.24, 29.26, 29.45, 29.51, 29.56, 29.59, 29.62, 32.29, 32.35, 32.40, 34.26, 34.38, 35.61, 36.72, 42.88, 53.75, 53.85, 53.94, 55.89, 55.95, 61.95, 62.15, 64.36, 64.54, 70.54, 172.58, 172.75, 173.76, 173.94.

##### Synthesis of PPMTP

PPMTP copolymers were prepared according to analogous procedures used for synthesis of PEG‐PPMTP block copolymers except that no MeO‐PEG5K‐OH was added to the feed mixtures and 1:1 TDDP/MDEA molar ratio was maintained in all monomer feeds. The monomer ratios and isolated polymer yields are shown in Table 1. The formed PPMTP terpolymers are abbreviated as *x*%P in which *x*% indicates molar percentage of PDL units versus (PDL+TDDP) units in the polymer chains. PPMTP without PDL: ^1^H NMR (CDCl_3_; ppm) 1.18 (br., 10H), 1.27 (br., 2H), 1.65 (br., 4H), 1.94 (t/br., 4H), 2.34 (s, 3H), 2.52 (t, 4H), 2.66 (t, 4H), 2.70 (t, 4H), 2.86 (d/br., 4H), 4.17 (t, 4H); ^13^C NMR (CDCl_3_; ppm) 23.87, 32.32, 32.37, 35.62, 36.73, 42.88, 53.77, 53.88, 55.90, 62.14, 172.60. PPMTP with PDL: ^1^H NMR (CDCl_3_; ppm) 1.19 (br.), 1.25 (br.), 1.64 (br.), 1.95 (t/br.), 2.31 (m), 2.34 (s), 2.52 (t), 2.67 (br.), 2.70 (t), 2.86 (d/br.), 4.06 (m), 4.17 (m); ^13^C NMR (CDCl_3_; ppm) 23.87, 24.92, 25.01, 25.90, 25.93, 28.60, 28.65, 29.15, 29.25, 29.28, 29.48, 29.52, 29.58, 29.60, 29.63, 32.30, 32.35, 34.27, 34.39, 35.58, 36.70, 42.88, 53.72, 53.79, 53.88, 55.89, 55.95, 61.94, 62.15, 64.38, 64.58, 172.56, 172.73, 173.78, 173.97.

##### Synthesis of MAL‐PEG‐PPMTP

MAL‐PEG‐PPMTP with 30% PDL and 41 wt% PEG were synthesized according to similar procedures for producing PEG‐PPMTP‐30% PDL (PEG30%P) except that maleimide‐terminated MAL‐PEG5K‐OH was used to replace MeO‐PEG5K‐OH in the monomer feed. The resultant MAL‐PEG‐PPMTP‐30% PDL (MAL‐PEG30%P) possessed same chain structures as in PEG30%P but different end groups and molecular weight. The presence of maleimide terminal groups was supported by a small distinct proton resonance of the polymer at 6.5 ppm. In contrast, all PEG‐PPMTP copolymers exhibited a small proton absorption at 3.4 ppm due to the methoxy (—OCH_3_) end groups. The synthesized MAL‐PEG30%P had an *M*_n_ of 29 500 Da with polydispersity of 3.1. This polymer was used for conjugation with AMD3100 to fabricate AMD3100‐conjugated PEG30%P nanoparticles.

##### NP Synthesis

NPs were fabricated using nanoprecipitation. In a typical synthesis, a selected polymer was dissolved in tetrahydrofuran (THF) and added dropwise into deionized water under stirring. After 30 min, the solution was transferred to a dialysis membrane (MWCO 3500 Da, ThermoFisher) and dialyzed against sodium acetate buffer (pH 5.2) for overnight. Prior to use, DNA at the amount calculated based on N/P ratio was added to the NP suspension and incubated for 30 min. For surface modification, AMD3100 was activated according to a recent report,[qv: 12a] and added to the NP suspension before DNA was added.

##### DLS

DLS (Malvern Zetasizer) was used to detect the hydrodynamic size and surface charge of NPs. Each measurement was carried out in triplicate at 25 °C, and an average value was reported.

##### TEM

For TEM analysis, 10 µL aliquots of NPs were applied to carbon coated copper grids. A filter paper was used to absorb the NPs after 5 min. The samples were then negatively stained by applying 10 µL of 1% phosphotungstic acid. After 2 min, a filter paper was used to absorb the phosphotungstic acid solution. The grids were left at fume hood until completely dried and then visualized by using a JEOL 1230 transmission electron microscope (JEOL Ltd., Japan) at 100 kV.

##### SEM

NPs after lyophilization were mounted on carbon tape and sputter‐coated with gold, under vacuum, in an argon atmosphere, using a sputter current of 40 mA (Dynavac Mini Coater, Dynavac, USA). SEM imaging was carried out with a Philips XL30 SEM using a LaB electron gun with an accelerating voltage of 10 kV. The mean diameter and size distribution of the particles were determined by image analysis using image analysis software (ImageJ, National Institutes of Health). These microscopy images were also used to assess particle morphology.

##### In Vitro Gene Transfection

231BR or U87 cells were plated at a density of 2.5 × 10^4^ cells per well in 48‐well plates. The plasmid encoding luciferase pGL4.13 (Promega) was used as the model gene to be encapsulated into NPs in different formulations for evaluating in vitro gene transfection. Control transfection using Lipofectamine 2000 (Lip2k, ThermoFisher) was performed per the standard protocol described in the manufacturer's manual. Briefly, Lip2k was mixed with DNA at a v/m ratio of 2.5, and the complexes were added to cells after incubation at room temperature for 20 min. For evaluation of NPs, NPs loaded with 0.5 ug DNA were prepared as described above and added to each well. After 24 h, medium containing Lip2k or NPs was replaced with fresh cell culture medium. Luciferase assays were performed 48 h post‐treatment when the cells were lysed with 100 µL reporter lysis buffer (Promega). After a freeze‐thaw cycle, the cell lysate was collected. After centrifugation at 15 000 rpm for 5 min, 20 µL of the supernatant was collected for luciferase assay using the Luciferase Assay Reagent (Promega) according to the standard protocol described in manufacturer's manual. An additional 20 µL was collected and used to quantify protein concentration using a BCA protein assay kit (ThermoFisher). The luciferase signal was divided by the amount of total protein for normalization.

##### In Vitro Cytotoxicity Evaluation

Cells were plated at a density of 4000 cells in 100 µL per well in 96‐well plates. After overnight incubation, cells were treated with NPs at various concentration. After 72 h, cell proliferation was quantified using the standard dimethyl thiazolyl diphenyl tetrazolium salt (MTT) assay according to a recent report.[qv: 23a]

##### Pharmacokinetics Study

Mice (*n* = 4) received intravenous administration of IR780‐loaded NPs. Blood samples were collected at 0.5, 1, 2, 4, 8, 12, 24, and 48 h after injection. The plasma IR780 concentration was quantified based on IR780 fluorescence and plotted with time.

##### Expression and Purification of Promelittin

Promelittin was expressed in CHO cells and purified over Ni Superflow Resin (Takarabio). Purity was confirmed by SDS‐PAGE prior to use. Concentrations were determined by a BCA assay (ThermoFisher).

##### In Vivo Tumor Models

All animal experiments were approved by the Yale University Institutional Animal Care and Utilization Committee. Female nude mice (NCr nu/nu) were purchased from Charles River Laboratories, and maintained in a pathogen‐free facility. Mouse xenografts were established according to a recent report.[qv: 9b] Briefly, 5 to 6 week old nude mice were anesthetized and firmly secured with front paws extended above the head. About 250 000 231BR cells in 0.1 mL PBS were loaded into a syringe with a 26‐G needle. After inserting the needle into the second intercostal space 3 mm to the left of the sternum with a depth of ≈6 mm, cells were injected slowly over 20–30 s.

##### Flow Cytometry and Immunochemistry

To characterize the expression of iRFP, 231BR cells were treated with iRFP720‐loaded NPs. After 48 h incubation, the cells were collected and analyzed using a BD FACSCalibur flow cytometer. To characterize the expression of CXCR4 in vitro, 231BR cells were collected, incubated with a primary antibody targeting CXCR4 (1:200, Novus NB100‐74396) and then an Alexa Fluor 488 conjugated secondary antibody (Thermo Scientific), and subjected to flow cytometry analysis. To characterize the expression of CXCR4 in tumors, the brains containing 231BR tumors were collected, fixed in 10% formalin solution at 4 °C overnight, and sectioned into slices in thickness of 6 µm. The sections were permeabilized with 0.1% Triton, blocked by 5% goat serum in Tris‐buffered saline with 0.1% Triton (TBST), and incubated with the anti‐CXCR4 antibody, followed with an Alexa Fluor 488 conjugated secondary antibody. Cell nuclei were stained with 4′,6‐diamidino‐2‐phenylindole. Images were captured using a fluorescence microscope (Olympus Inc).

##### Fluorescent Imaging of NPs

Tumor bearing mice were randomly assigned into experimental groups (*n* = 3). Three weeks after tumor inoculation, IR780‐loaded NPs were administered intravenously through the tail vein. Dose for each group was adjusted according to the fluorescence intensity to ensure that each mouse received the same amount of dye. 24 h later, the mice were imaged by IVIS imaging system (Xenogen) with excitation wavelength of 745 nm and emission wavelength of 820 nm. Afterward, mice were euthanized, and major organs were isolated and imaged by IVIS using the same setting.

##### Determination of the Therapeutic Benefits

7 d after tumor inoculation, mice were randomly assigned into three experimental groups, which received intravenous administration of *proMel* NPs, control iRFP720 NPs, or PBS. NPs were given at 1 mg (200 ul NPs at a concentration of 500 ug mL^−1^ in PBS) per injection three times a week for two weeks. Tumor growth was monitored by in vivo bioluminescence imaging using the system of IVIS Spectrum. Mouse survival and body weight were recorded. When the mice died, the brains were collected, fixed, sliced, and subjected to the standard TUNEL and H&E staining.

##### Statistical Analysis

All data were collected in triplicate and reported as mean and standard deviation. Comparison between the groups were performed using a t‐test. One‐way ANOVA was used to analyze multiple comparisons by GraphPad Prism 7.0. *p < 0.05, **p < 0.01 and ***p < 0.001 were considered significant.

## Conflict of Interest

The authors declare no conflict of interest.

## Supporting information

Supporting Information

## References

[1] C. DeSantis , J. Ma , L. Bryan , A. Jemal , Ca‐Cancer J. Clin. 2014, 64, 52. 24114568 10.3322/caac.21203

[2] A. B. Lassman , L. M. DeAngelis , Neurol. Clin. 2003, 21, 1. 12690643 10.1016/s0733-8619(02)00035-x

[3] M. N. Tsao , D. Rades , A. Wirth , S. S. Lo , B. L. Danielson , L. E. Gaspar , P. W. Sperduto , M. A. Vogelbaum , J. D. Radawski , J. Z. Wang , M. T. Gillin , N. Mohideen , C. A. Hahn , E. L. Chang , Pract. Radiat. Oncol. 2012, 2, 210. 25925626 10.1016/j.prro.2011.12.004 PMC3808749

[4] D. P. Kodack , V. Askoxylakis , G. B. Ferraro , D. Fukumura , R. K. Jain , Cancer Cell 2015, 27, 163. 25670078 10.1016/j.ccell.2015.01.001 PMC4325273

[5] a) T. Patel , J. Zhou , J. M. Piepmeier , W. M. Saltzman , Adv. Drug Delivery Rev. 2012, 64, 701; 10.1016/j.addr.2011.12.006 PMC3323692 22210134

[6] a) J. Lu , P. S. Steeg , J. E. Price , S. Krishnamurthy , S. A. Mani , J. Reuben , M. Cristofanilli , G. Dontu , L. Bidaut , V. Valero , G. N. Hortobagyi , D. Yu , Cancer Res. 2009, 69, 4951; 19470768 10.1158/0008-5472.CAN-09-0099

[7] a) J. W. Han , Y. S. Yoon , Antioxid. Redox Signaling 2011, 15, 1799; 10.1089/ars.2010.3814 PMC3159104 21194386

[8] a) H. Lv , S. Zhang , B. Wang , S. Cui , J. Yan , J. Controlled Release 2006, 114, 100; 10.1016/j.jconrel.2006.04.014 16831482

[9] a) J. Zhou , J. Liu , C. J. Cheng , T. R. Patel , C. E. Weller , J. M. Piepmeier , Z. Jiang , W. M. Saltzman , Nat. Mater. 2012, 11, 82; 10.1038/nmat3187 PMC4180913 22138789

[10] a) P. Zarogoulidis , K. Darwiche , A. Sakkas , L. Yarmus , H. Huang , Q. Li , L. Freitag , K. Zarogoulidis , M. Malecki , J. Genet. Syndr. Gene Ther. 2013, *4*, 1000139; 10.4172/2157-7412.1000139 PMC3842193 24294541

[11] O. Frank , C. Rudolph , C. Heberlein , N. von Neuhoff , E. Schrock , A. Schambach , B. Schlegelberger , B. Fehse , W. Ostertag , C. Stocking , C. Baum , Blood 2004, 104, 3543. 15308565 10.1182/blood-2004-03-0852

[12] a) X. Guo , G. Deng , J. Liu , P. Zou , F. Du , F. Liu , A. T. Chen , R. Hu , M. Li , S. Zhang , Z. Tang , L. Han , J. Liu , K. N. Sheth , Q. Chen , X. Gou , J. Zhou , ACS Nano 2018, 12, 8723; 30107729 10.1021/acsnano.8b04787

[13] a) S. Liu , M. Yu , Y. He , L. Xiao , F. Wang , C. Song , S. Sun , C. Ling , Z. Xu , Hepatology 2008, 47, 1964; 18506888 10.1002/hep.22240

[14] T. Jiang , E. S. Olson , Q. T. Nguyen , M. Roy , P. A. Jennings , R. Y. Tsien , Proc. Natl. Acad. Sci. USA 2004, 101, 17867. 15601762 10.1073/pnas.0408191101 PMC539314

[15] a) D. Palmieri , J. L. Bronder , J. M. Herring , T. Yoneda , R. J. Weil , A. M. Stark , R. Kurek , E. Vega‐Valle , L. Feigenbaum , D. Halverson , A. O. Vortmeyer , S. M. Steinberg , K. Aldape , P. S. Steeg , Cancer Res. 2007, 67, 4190; 17483330 10.1158/0008-5472.CAN-06-3316

[16] J. Liu , Z. Z. Jiang , J. B. Zhou , S. M. Zhang , W. M. Saltzman , J. Biomed. Mater. Res., Part A 2011, 96A, 456. 10.1002/jbm.a.32994 PMC3080021 21171165

[17] a) X. F. Zhang , W. X. Tang , Z. Yang , X. G. Luo , H. Y. Luo , D. Gao , Y. Chen , Q. Jiang , J. Liu , Z. Z. Jiang , J. Mater. Chem. B 2014, 2, 4034; 10.1039/c4tb00439f 32261654

[18] J. S. Suk , Q. G. Xu , N. Kim , J. Hanes , L. M. Ensign , Adv. Drug Delivery Rev. 2016, 99, 28. 10.1016/j.addr.2015.09.012 PMC4798869 26456916

[19] D. M. Shcherbakova , V. V. Verkhusha , Nat. Methods 2013, 10, 751. 23770755 10.1038/nmeth.2521 PMC3737237

[20] X. Yu , X. Gou , P. Wu , L. Han , D. Tian , F. Du , Z. Chen , F. Liu , G. Deng , A. T. Chen , C. Ma , J. Liu , S. M. Hashmi , X. Guo , X. Wang , H. Zhao , X. Liu , X. Zhu , K. N. Sheth , Q. Chen , L. Fan , J. Zhou , Adv. Mater. 2018, 30, 1705383. 10.1002/adma.201705383 PMC5812013 29315863

[21] C. Y. Kim , M. Jeong , H. Mushiake , B. M. Kim , W. B. Kim , J. P. Ko , M. H. Kim , M. Kim , T. H. Kim , P. D. Robbins , T. R. Billiar , D. W. Seol , Gene Ther. 2006, 13, 330. 16195699 10.1038/sj.gt.3302658

[22] R. Trivedi , D. P. Mishra , Front. Oncol. 2015, 5, 69. 25883904 10.3389/fonc.2015.00069 PMC4382980

[23] a) Z. Chen , F. Liu , Y. Chen , J. Liu , X. Wang , A. T. Chen , G. Deng , H. Zhang , J. Liu , Z. Hong , J. Zhou , Adv. Funct. Mater. 2017, 27, 1703036; 29755309 10.1002/adfm.201703036 PMC5939593

[24] I. Zafir‐Lavie , S. Sherbo , H. Goltsman , F. Badinter , E. Yeini , P. Ofek , R. Miari , O. Tal , A. Liran , T. Shatil , S. Krispel , N. Shapir , G. A. Neil , I. Benhar , A. Panet , R. Satchi‐Fainaro , J. Controlled Release 2018, 291, 80. 10.1016/j.jconrel.2018.10.017 30342077

[25] Y. Chen , X. Gou , D. K. Kong , X. Wang , J. Wang , Z. Chen , C. Huang , J. Zhou , Oncotarget 2015, 6, 32575. 26416452 10.18632/oncotarget.5331 PMC4741713

