# Supplementary material for: Targeted Delivery of Secretory Promelittin via Novel Poly(lactone‐co‐β‐amino ester) Nanoparticles for Treatment of Breast Cancer Brain Metastases
Source: Adv Sci (Weinh). 2020 Jan 19;7(5):1901866. doi: 10.1002/advs.201901866 (PMC7055583; doi:10.1002/advs.201901866)
Supplement: Supplementary file 1 — Supporting Information [file ADVS-7-1901866-s001.pdf]

Copyright WILEY-VCH Verlag GmbH & Co. KGaA, 69469 Weinheim, Germany, 2020.

ADVANCED SCIENCE
Open Access

Supporting Information

for *Adv. Sci.,* DOI: 10.1002/advs.201901866

Targeted Delivery of Secretory Promelittin via Novel Poly(lactone-*co*-β-amino ester) Nanoparticles for Treatment of Breast Cancer Brain Metastases

Yu Zhou, Shenqi Zhang, Zeming Chen, Youmei Bao, Ann T. Chen, Wendy C. Sheu, Fuyao Liu, Zhaozhong Jiang, and Jiangbing Zhou**

Copyright WILEY-VCH Verlag GmbH & Co. KGaA, 69469 Weinheim, Germany, 2019.

Supporting Information

Targeted Delivery of Secretory Pro-melittin via Novel Poly(lactone-co-*β*-amino ester) Nanoparticles for Treatment of Breast Cancer Brain Metastases

Yu Zhou, Shenqi Zhang, Zeming Chen, Youmei Bao, Ann T. Chen, Wendy C. Sheu, Fuyao Liu, Zhaozhong Jiang, Jiangbing Zhou**

Preparation of TDDP monomer

Experimental procedures

Typically, ethyl acrylate (12.19 g, 121.8 mmol) and 4,4'-trimethylenedipiperidine (12.81 g, 60.9 mmol) were dissolved in diphenyl ether solvent (25.00 g). The resultant mixture was stirred initially at ambient temperature for 1 h, and subsequently at 50 °C for 24 h. Afterwards, NMR analyses indicated that the substrates were completely converted to form highly pure diethyl 3,3'-(4,4'-trimethylenedipiperidine-1,1'-diyl)dipropionate monomer (TDDP, 50 wt% in diphenyl ether).

TDDP: ^{1}H NMR ($CDCl_3$; ppm) 1.18 (br.), 1.23 (t), 1.27 (br.), 1.62 (br., 4H), 1.93 (t/br., 4H), 2.48 (t, 4H), 2.66 (t, 4H), 2.85 (d/br., 4H), 4.11 (q, 4H); total integration from 1.18 to 1.27 ppm equals to 18 H; ^{13}C NMR ($CDCl_3$; ppm) 14.2, 23.9, 32.4, 32.5, 35.6, 36.8, 53.8, 54.0, 60.2, 172.6.

Synthesis reaction and structural characterization

TDDP monomer was synthesized via Michael addition reaction between ethyl acrylate and 4,4'-trimethylenedipiperidine under mild reaction conditions (Scheme S1). This reaction is mildly exothermic. At 2:1 acrylate/diamine molar ratio and given a sufficient reaction time (24 h), the reaction proceeds quantitatively to generate high purity TDDP, which can be directly used for polymerization reactions without further purification. The molecular structure of TDDP was confirmed by both ^{1}H and ^{13}C NMR spectroscopy analyses (detailed assignments of the NMR resonances are described in Figure S1).

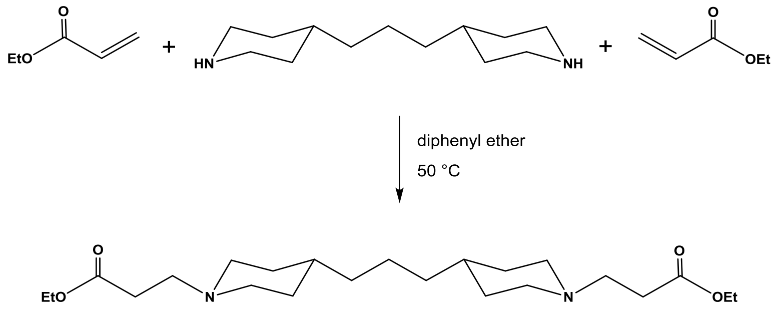

Scheme S1. Synthetic method to prepare TDDP monomer

A

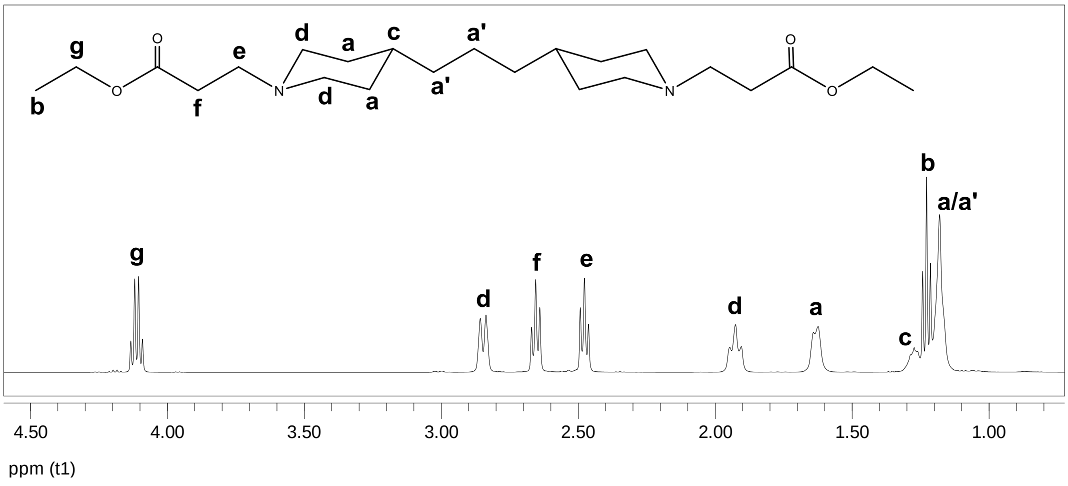

B

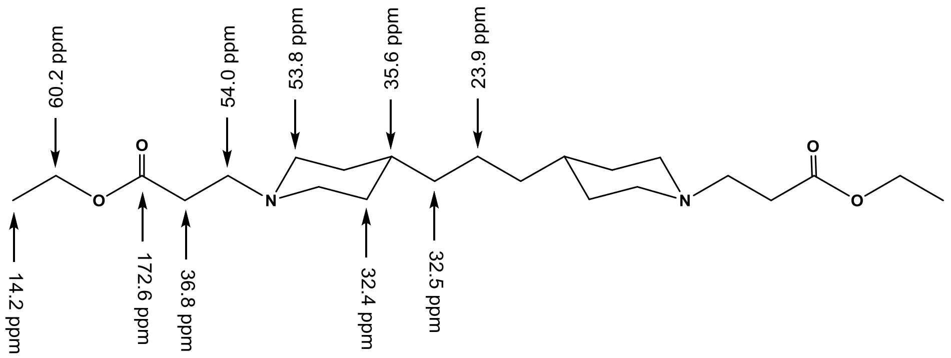

Figure S1. Structural assignments for (A) the proton and (B) carbon-13 NMR resonances of TDDP monomer

Structural analysis of PPMTP and PEG-PPMTP by proton and carbon-13 NMR spectroscopy

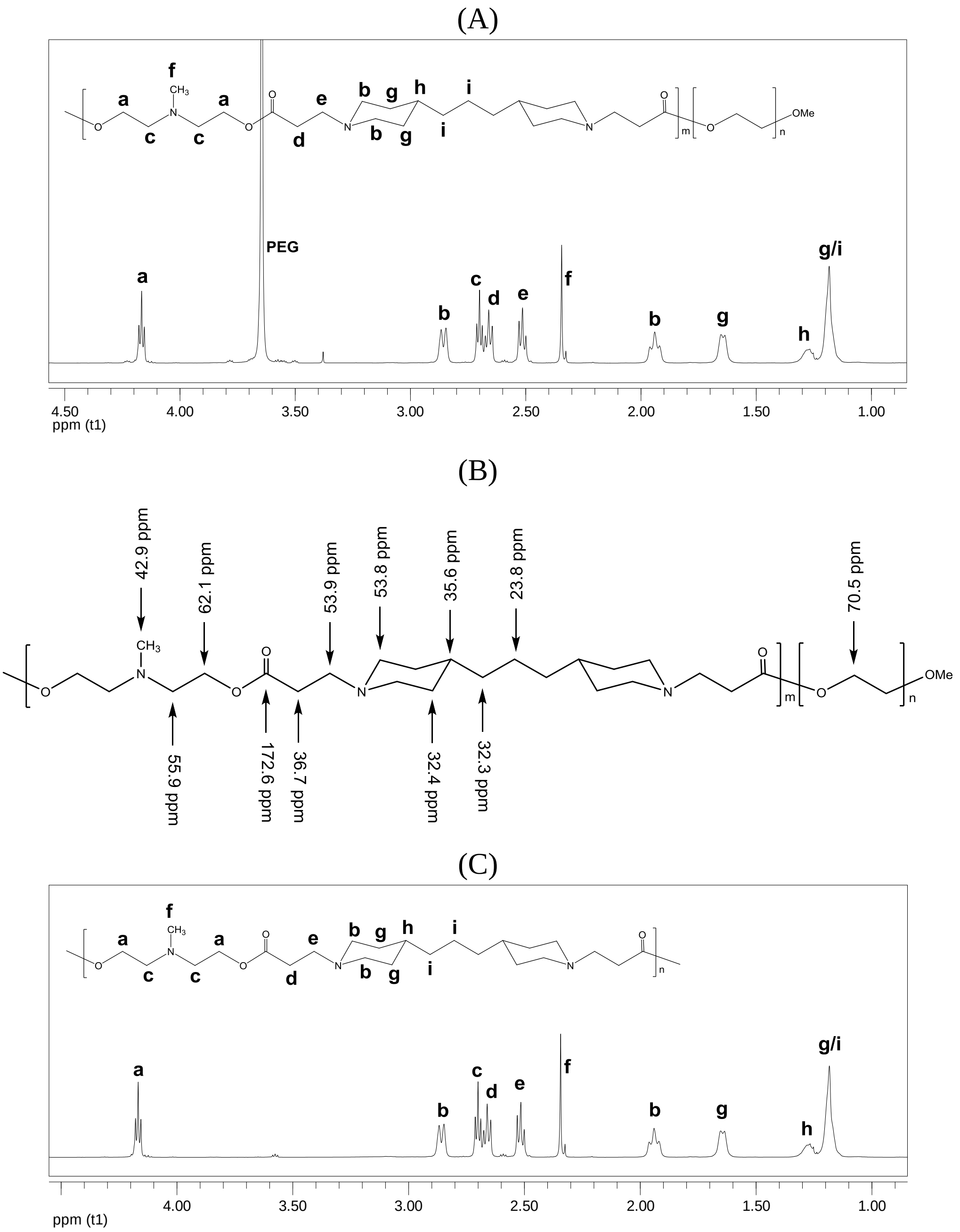

Figure S2. Structural analysis of PEG-PPMTP and PPMTP copolymers. Structural assignments for (A) the proton and (B) major carbon-13 NMR resonances of PEG-PPMTP with 0% PDL (PEG0%P) block copolymer, and for (C) the proton absorptions of PPMTP with 0% PDL (0%P) copolymer. Chloroform-*d* was used as the solvent.

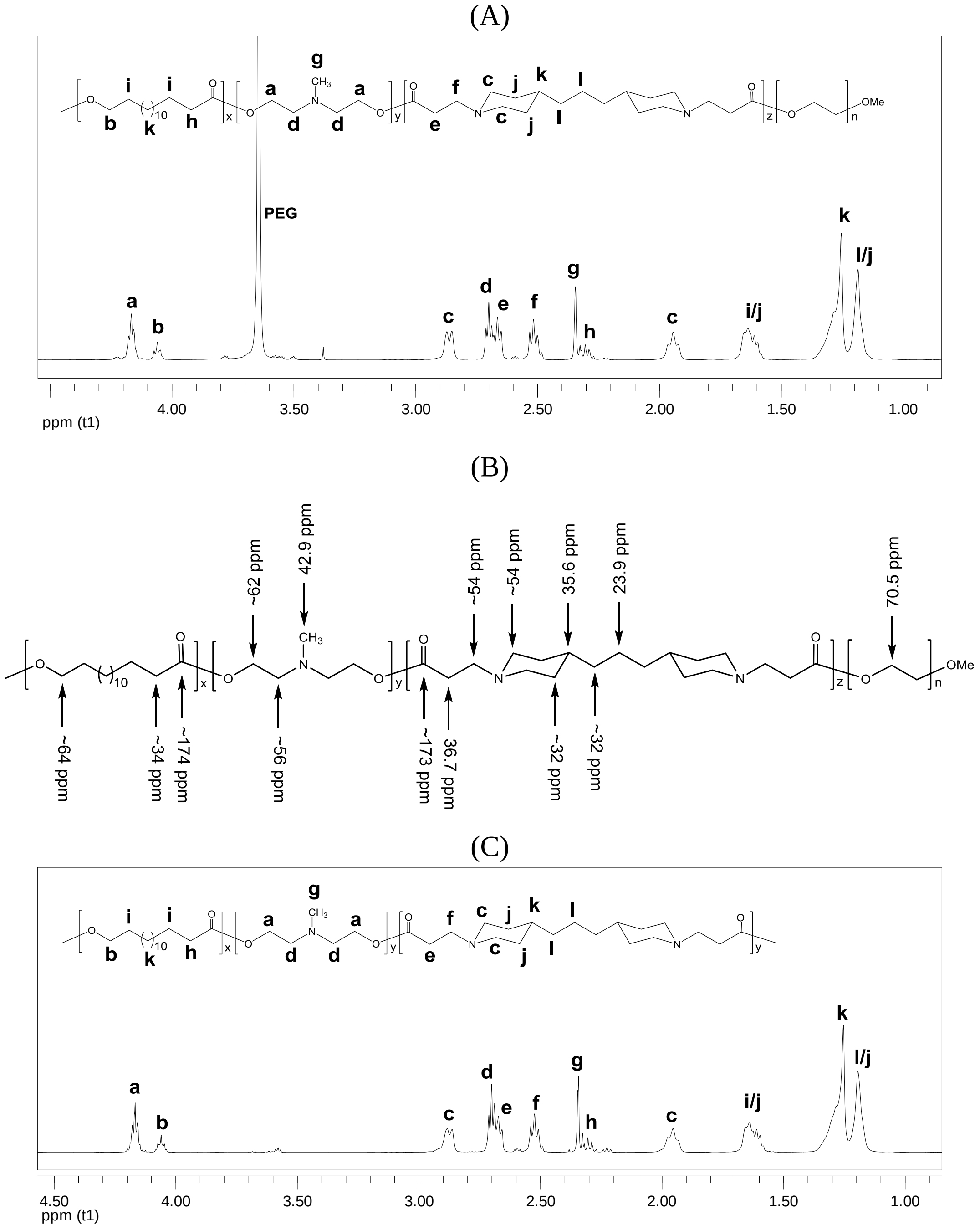

Figure S3. Structural assignments for (A) the proton and (B) major carbon-13 NMR resonances of PEG-PPMTP block copolymer (PEG40%P), and for (C) the proton absorbances of PPMTP copolymer (40%P).

(A)

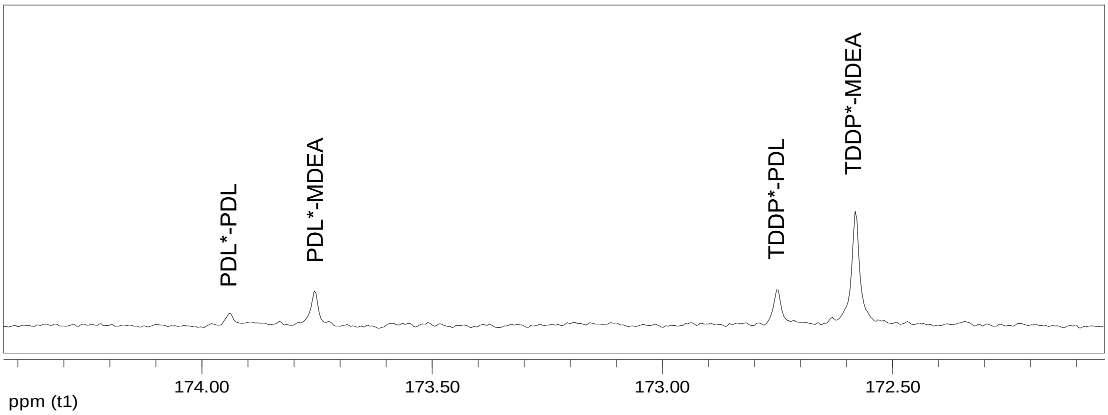

(B)

sample	PDL*-PDL		PDL*-MDEA		TDDP*-PDL		TDDP*-MDEA	
	meas. [a]	calc. [b]	meas. [a]	calc. [b]	meas. [a]	calc. [b]	meas. [a]	calc. [b]
PEG20%P	0.02	0.01	0.10	0.11	0.10	0.11	0.78	0.77
PEG40%P	0.07	0.07	0.18	0.19	0.18	0.19	0.57	0.55
40%P	0.06	0.07	0.18	0.19	0.18	0.19	0.57	0.55

a. Measured by carbon-13 NMR spectroscopy.
b. Calculated for random poly(PDL-*co*-MDEA-*co*-TDDP) chains. Abundance of PDL-PDL diad = $f_P \times f_P$; abundance of PDL-MDEA diad = $f_P \times (2 \times f_M)$; abundance of TDDP-PDL diad = $(2 \times f_T) \times f_P$; abundance of TDDP-MDEA diad = $(2 \times f_T) \times (2 \times f_M)$. The symbols f_P, f_M, f_T represent, respectively, molar fractions of PDL, MDEA, and TDDP units in the copolymer chains.

Figure S4. (A) Carbonyl carbon-13 absorbances of different diads in PEG40%P (similar resonances were observed in 40%P), and (B) diad distributions in the polyester blocks of PEG-PPMTP copolymers and in a representative PPMTP (40%P).

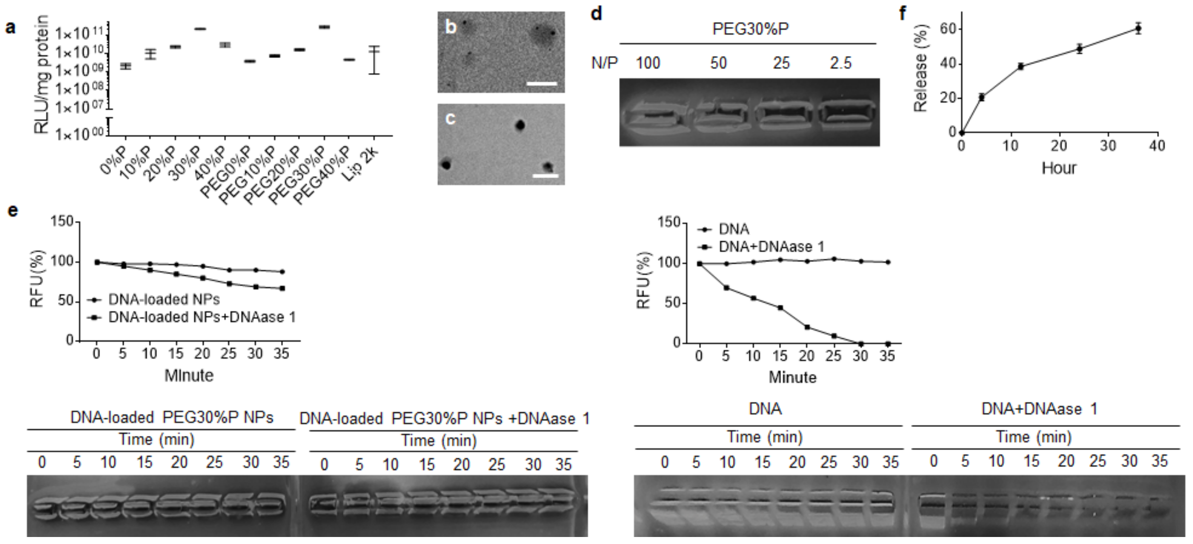

Figure S5. Characterization of polymeric nanoparticles for gene delivery. a, Gene delivery efficiency of the indicated polymers on U87 cells. b,c, Representative TEM images of 30%P NPs (b) and PEG30%P NPs (c). Scale bar: 200 nm. d, Characterization of DNA encapsulation by gel retardation assay. DNA-loaded NPs were prepared at the indicated N/P ratios, mixed with DNA loading buffer, and subjected to analysis using 1.5% gel. DNA migration was observed using a UV transilluminator (Cell Biosciences Fluor ChemQ). e, Protection of DNA from enzymatic degradation by the indicated NPs. DNA-loaded NPs (left) and naked DNA (right) were subjected to DNase degradation at 37℃ for indicated time. Residual DNA was quantified used PicoGreen (Invitrogen). The values are expressed as a percentage of the fluorescence obtained at time 0 min. In the bottom panels, below each graph, the corresponding agarose gel electrophoresis is shown. f, Release of DNA vs. time from PEG30%P NPs.

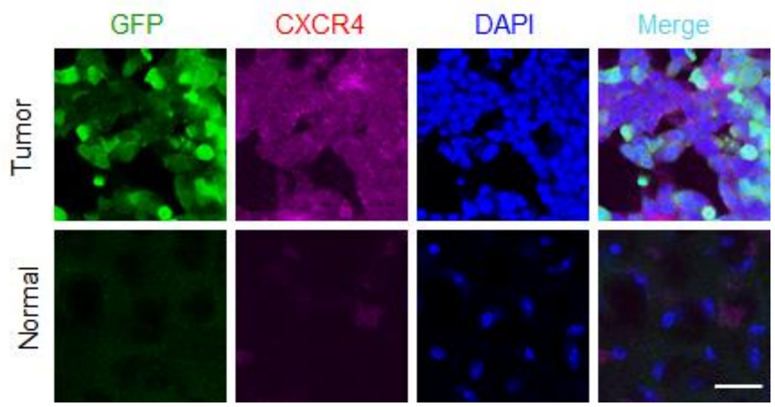

Figure S6. Representative images of the expression of CXCR4 in the region with tumor (GFP) and without tumor. Scale bar: 20 μm.

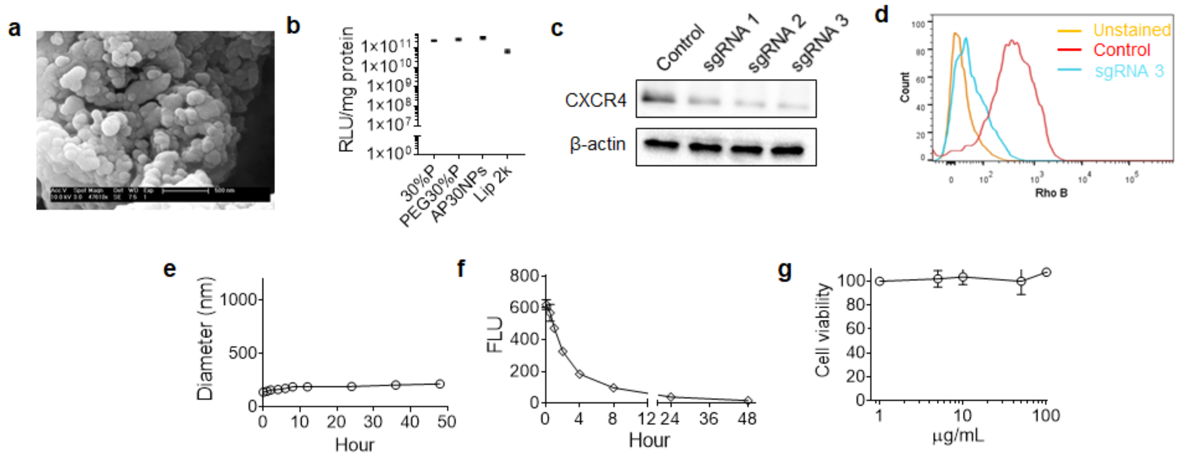

Figure S7. Characterization of AP30NPs. a, A representative SEM image of AP30NPs. b, Gene delivery efficiency of the indicated polymers on 231BR cells. c, Western Blot analysis of the expression of CXCR4 in 231BR cells after transduction of lentivires containing Cas9 and the indicated sgRNA. Sequences for the sgRNAs are as follows: sgRNA 1: TACACCGAGGAAATGGGCTC; sgRNA 2: GAAGAAACTGAGAAGCATGA; sgRNA 3: CGTGATGACAAAGAGGAGGT. d, Flow cytometry analysis of the uptake of Rhodamine b (Rho B)-loaded NPs by cells with down-regulation of CXCR4 by sgRNA3. e, Change of the diameter of AP30NPs versus time in serum-containing medium. f, Plasma concentrations of IR780 versus time in mice after intravenous administration of IR780-loaded AP30NPs. g, Cytotoxicity of AP30NPs on 231BR cells. Toxicity was given as the percentage of viable cells remained after treatment for three days, compared to the control vehicle treated cells.

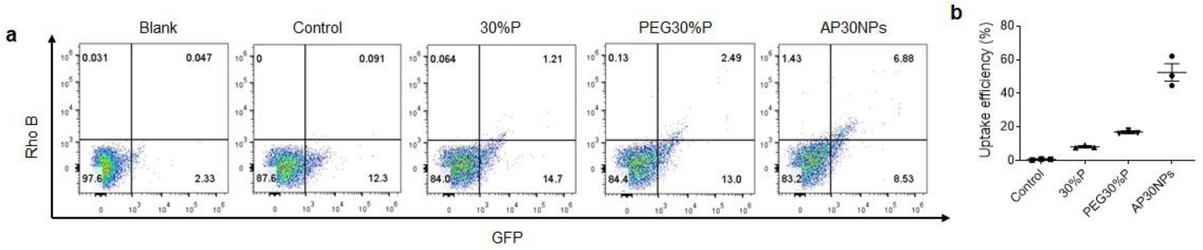

Figure S8. Characterization of uptake of NPs in tumors after intravenous administration. a, Flow cytometry analysis of Rho B-positive cells in the brains isolated from mice received the indicated treatment. b, Quantification of the uptake efficiency in tumor cells, where were identified based on GFP expression.

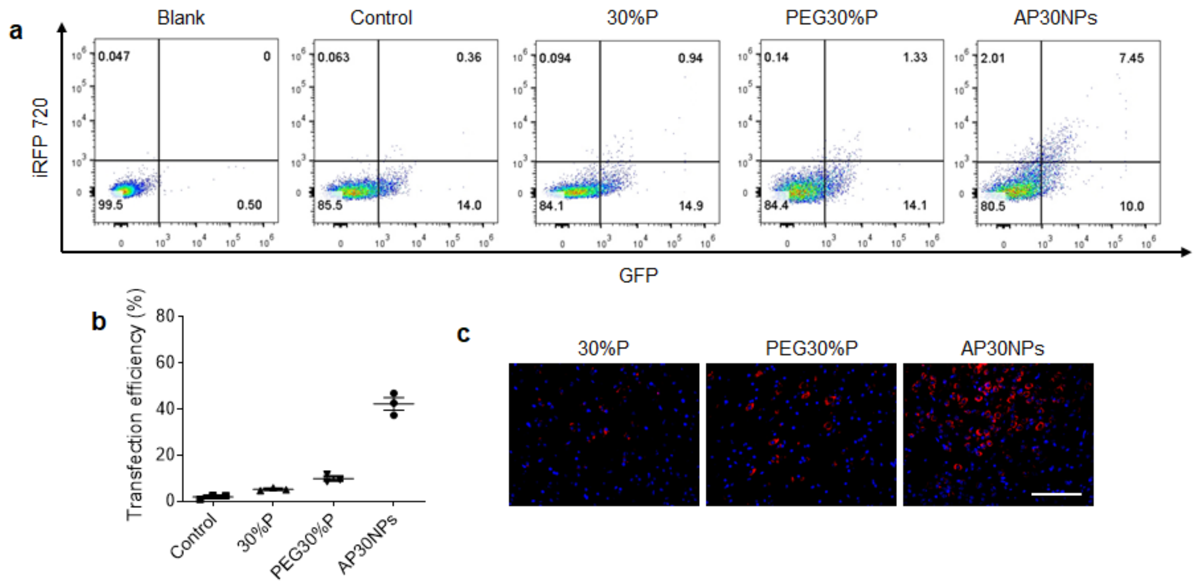

Figure S9. Characterization of transfection of tumors after intravenous administration of NPs loaded with iRFP720 DNA. a, Flow cytometry analysis of iRFP720-positive cells in the brains isolated from mice received treatment of the indicated NPs loaded with iRFP720 DNA. b, Quantification of iRFP720-positive cells within tumors, where were identified based on GFP expression. c, Representative images of iRFP720-positive cells in tumors isolated from mice received treatment of the indicated NPs loaded with iRFP720 DNA. Scale bar: 30 μm.

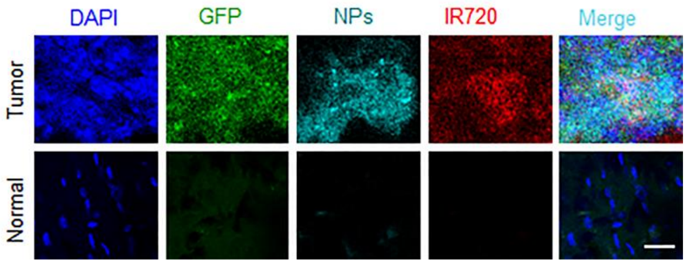

Figure S10. Confocal microscopic analysis of the distribution of AP30NPs, which were identifed based on the fluorescence of Rho B encapsulated in the NPs, and transfected cells, which were identifed based on the expression of IR720, after intravenous adminstration. Scale bar: 30 μm.

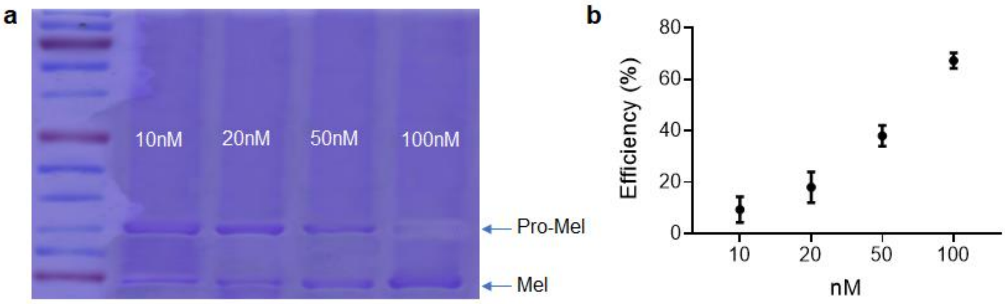

Figure S11. Characterization of cleavage efficiency of pro-melittin by MMP-2. a, Gel electrophoresis analysis of pro-melittin proteins after incubation with MMP-2 at the indicated concentrations. Incubation was performed 37 ^{o}C for 30 minutes. b, Quantification of MMP-2 cleavage efficiency based on gel electrophoresis analysis.

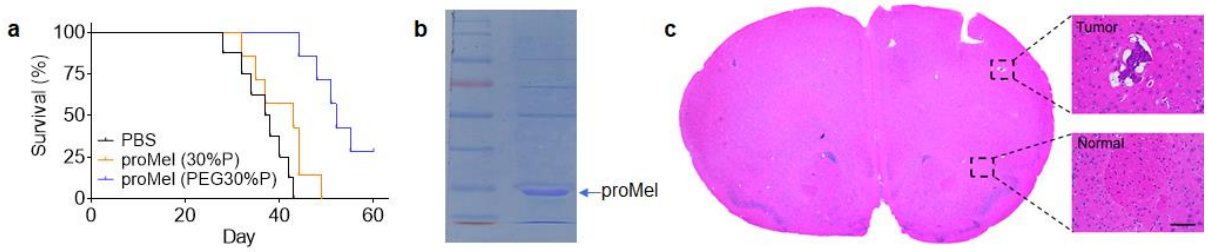

Figure S12. Characterization the NP-based therapy in mice. a, Kaplan-Meier survival curves of tumor-bearing mice received the indicated treatments. b, Gel electrophoresis analysis of pro-melittin protein in tumors. Tumors were isolated from the brain of mice treated with *proMel* NPs, homogenized, incubated through His 60 Ni Superflow Resin (TakaRa, USA), and subjected to gel electrophoresis analysis. c, A representative image of H&E staining of the brain isolated from a mouse treated with *proMel* NPs.

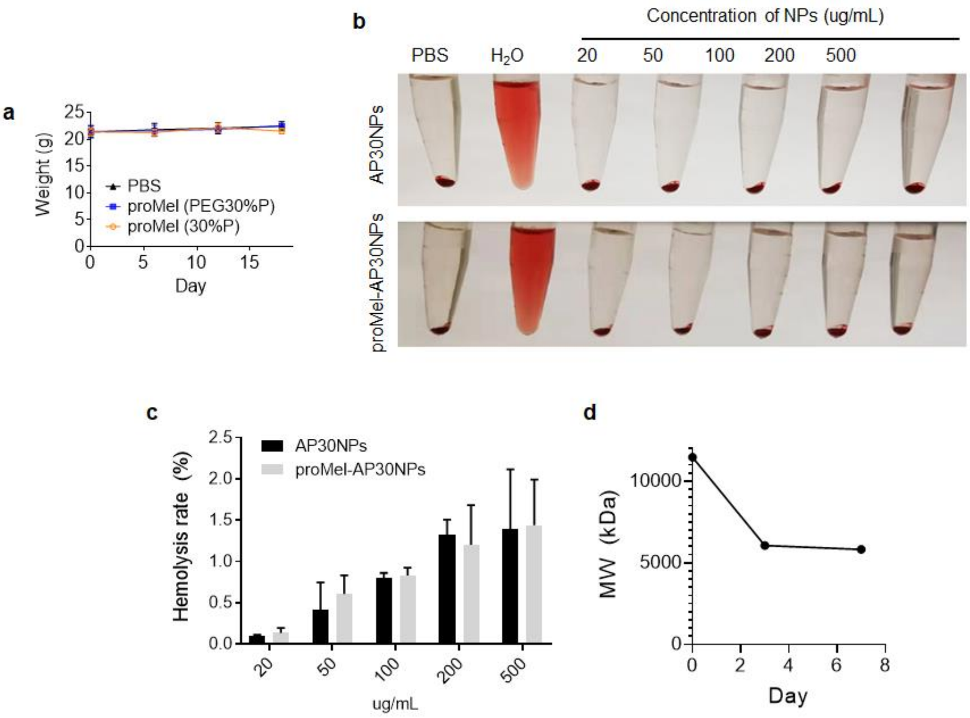

Figure S13. Characterization of NPs and polymers. a, Change of body weight with time in mice received the indicated treatments. b, Representavie images of red blood cells incubated with the indicated polymers. c, Quantification of the hemolytic activity of the indicated NPs. d, Change of molecular weight of PPMTP with time after incubation in PBS (pH 7.4).

Hemolytic activity was determined by measuring the release of hemoglobin from erythrocytes after incubation with AP30NPs or proMel-AP30NPs. Briefly, blood was collected from mice and added to eppendorf tubes containing PBS. After centrifugation and wash with saline, red blood cells (RBCs) were obtained and diluted to 10 mL saline. RBC suspension of 0.4 mL was then incubated with the tested NPs at concentrations of 0, 20, 50, 100, 200, 500 ug/mL at 37 ºC with gentle shaking. RBC treated with PBS (0% lysis) and water (100% lysis) were used a negative control and a positive control, respectively. After 1 hour, the samples were centrifuged for 5 min at 3000 rpm. Absorbance of the supernatant was determined by a UV-vis spectrophotometer at 545 nm. Hemolysis rate was calculated using the following equation: Hemolytic rate (%) = $[(A_{\text{sample}} - A_{\text{PBS}})/(A_{\text{water}} - A_{\text{PBS}})] \times 100\%$.

a. DNA Sequence of *proMel*
cctcttggacttgctggtggaattggcgccgtgctgaaggtgctgacaacaggactgcctgctctgatcagctggatcaagcggaaga gacagcagcctctgggactcgctgga
b. Sequence of *proMel* expression product
DAAQPARRARRTKLPLGLAGGIGAVLKVLTTGLPALISWIKRKRQQPLGLAGARGGP EQKLISEEDLNSAVDHHHHHH
Black: sequences for vector pSecTag2; Green: MMP-2 cleavable sequences; Red: melittin.

Figure S14. Sequence of artificial gene *proMel* and its expression product.
